# A Novel Inflammatory Marker for the Diagnosis of Hashimoto’s Thyroiditis: Platelet-Count-to-Lymphocyte-Count Ratio

**DOI:** 10.3390/diseases11010015

**Published:** 2023-01-22

**Authors:** Eray Erge, Cagri Kiziltunc, Sumeyye Buse Balci, Burcin Meryem Atak Tel, Satilmis Bilgin, Tuba Taslamacioglu Duman, Gulali Aktas

**Affiliations:** Department of Internal Medicine, Abant Izzet Baysal University Hospital, 14200 Bolu, Turkey

**Keywords:** Hashimoto’s thyroiditis, inflammation, platelet-to-lymphocyte ratio

## Abstract

Background: Hashimoto’s thyroiditis (HT) is a chronic autoimmune thyroiditis that causes systemic inflammation in the body, leading to hypothyroidism and an enlargement of the thyroid gland. Objectives: This study aims to reveal whether there is a relationship between Hashimoto’s thyroiditis and the platelet-count-to-lymphocyte-count ratio (PLR), which is used as a new inflammatory marker. Methods: In this retrospective study, we compared the PLR of the euthyroid HT group and the hypothyroid-thyrotoxic HT group to the controls. We also evaluated the values of thyroid-stimulating hormone (TSH), free T4 (fT4), C-reactive protein (CRP), aspartate transaminase (AST), alanine transaminase (ALT), white blood cell count, lymphocyte count, hemoglobin, hematocrit, and platelet count in each group. Results: The PLR of the subjects with Hashimoto’s thyroiditis was found to be significantly different from the control group (*p* < 0.001), with the rankings as follows: hypothyroid-thyrotoxic HT 177% (72–417) > euthyroid HT 137% (69–272) > control group 103% (44–243). In addition to the increased PLR values, an increase in CRP values was also observed, revealing a strong positive correlation between the PLR and CRP in the HT patients. Conclusion: In this study, we found out that the PLR was higher in the hypothyroid-thyrotoxic HT and euthyroid HT patients than in a healthy control group.

## 1. Introduction

Hashimoto’s thyroiditis (HT) is a disease characterized by the chronic autoimmune inflammation of the thyroid tissue, which was defined by Dr. Hakaru Hashimoto in 1912 as a result of the histopathological evaluation of the thyroid tissue of patients with histologically different features [1]. HT, also called chronic lymphocytic thyroiditis, is one of the most common reasons for goiter hypothyroidism [2]. The general characteristics of HT include diffuse lymphomononocytic cell infiltration; thyroid gland hypertrophy; abnormal thyroid functions; morphological changes in thyroid sonography; increased titers of thyroid autoantibodies, anti-thyroid peroxidase (Anti-TPO), and anti-thyroglobulin (Anti-TG); decreased number of thyroid follicles; and Hurthle cells with granular-pink cytoplasm [3]. HT, which is an autoimmune thyroid disease with widespread lymphocytic infiltration in the thyroid gland, is a common disease with a rate of 2% in the population, and its frequency is gradually rising [4].

The thyroid gland is mainly responsible for producing tetraiodothyronine (thyroxine-T4) and, to a lesser extent, triiodothyronine (T3). It is the largest endocrine organ and weighs approximately 15–20 g in adults. The secretion of thyrotropin-releasing hormone (TRH) from the hypothalamus and thyroid-stimulating hormone (TSH) from the pituitary constitute the first steps in regulating thyroid gland functions. About 1/4 of basal metabolism in most tissues is regulated by thyroid hormones. Changes in the circulating thyroid hormone levels in the pituitary will result in an increase or a decrease in TSH release, leading to either hyperthyroidism or hypothyroidism.

Thyroiditis is the inflammation of the thyroid gland. Hashimoto’s thyroiditis is a type of thyroiditis that is caused by a dysfunction in the immune system. In patients with Hashimoto’s thyroiditis, the immune system attacks the cells in the thyroid gland over a long period, causing inflammation. In this phase, an increased release of thyroid hormones from thyroid follicles to the bloodstream may cause thyrotoxicosis. The thyroid cells gradually disappear due to this disorder, and eventually, thyroid gland failure develops as there are no cells to produce hormones. This phase is characterized by hypothyroidism. Thyroiditis is a group of inflammatory diseases of the thyroid gland. It is classified as either infectious, subacute (De Quervain’s thyroiditis), Riedel’s, drug-induced, silent, postpartum, or autoimmune thyroiditis (also known as chronic thyroiditis or Hashimoto’s thyroiditis). Hashimoto’s thyroiditis is an autoimmune thyroid condition. Patients with Hashimoto’s thyroiditis usually have antibodies to thyroglobulin in their serum. Auto-antibodies also develop against thyroid surface antigens and a second colloid antigen. The influences of both the antibodies acting against microsomes and specially programmed T cells may damage the thyroid tissues and result in myxedema. In the hypothyroid phase of Hashimoto’s thyroiditis, the TSH level rises following the physiological response (due to the diminished negative feedback effect of thyroid hormones). TSH regulates thyroid hormone synthesis in the thyroid gland.

The prevalence of Hashimoto’s thyroiditis varies depending on various factors, such as the criteria used for diagnosing the disease, the environmental conditions of the region where the study is conducted, gender, and age. The incidence of Hashimoto’s thyroiditis has been increasing in recent years, possibly due to the use of more accurate diagnostic tests and the increased consumption of dietary iodine. In countries with a high iodine intake, Hashimoto’s thyroiditis is the most common cause of hypothyroidism. With the elimination of iodine deficiency, the prevalence of goiter due to autoimmune thyroiditis is increasing [5]. Hashimoto’s thyroiditis is 15–20 times more common in women than in men, and it is more common in people between the ages of 30 and 50 years, although it can occur at any age, including in childhood. Hashimoto’s thyroiditis is one of the most common autoimmune diseases, affecting more than 2% of the general population and contributing to morbidity, especially in women [6]. Autoimmune thyroiditis may occur due to genetic and non-genetic (environmental) factors. Depending on the genetic factors, the risk of developing Hashimoto’s thyroiditis in individuals with a family history of thyroid disease is higher than in the general population [7]. The incidence of this disease in the first-degree relatives of individuals with Hashimoto’s thyroiditis is about 18–33%. High levels of anti-thyroid peroxidase (Anti-TPO) and anti-thyroglobulin (Anti-TG) antibodies indicate that this thyroid disease occurs due to a disorder in the immune system. However, these antibodies can be found to be present in high titers in 10% of individuals without a thyroid disease in the general population. Anti-TPO antibodies are 95% positive in Hashimoto’s thyroiditis and 85% positive in Graves’ disease. There is no relationship between positive antibody titers or levels and disease severity. However, the rate of thyroid antibody positivity in individuals with Hashimoto’s thyroiditis has been found to be higher than the average rate in the general population [8].

Various factors can lead to the secretion of cytokines in the serum in Hashimoto’s thyroiditis. Cytokine secretion can vary in amount depending on the severity of the inflammatory response. Nuclear factor kappa B (NF-kB) is one of the most important of these factors. NF-kB is a transcription factor that activates inflammatory genes in conjunction with poly ADP-ribose polymerase-1 (PARP-1) protein. NF-kB acts through its interactions with other regulatory molecules, rather than functioning independently, in patients with Hashimoto’s thyroiditis.

As mentioned above, Hashimoto’s thyroiditis is an autoimmune disease caused by T-cell-mediated autoimmunity, which results from the harmful interaction between genetic and environmental factors. Dietary iodine intake, bacterial and viral infections, cytokine therapy, and pregnancy are among the environmental factors that may contribute to the development of Hashimoto’s thyroiditis [9]. This disease tends to run in families, with siblings being up to 20 times more likely to develop it and monozygotic twins having a probability of 30–60% of having the same disease. It is also more common in people with Down syndrome and Turner syndrome and has a weak association with HLA DR3 and HLA DR5. Cytotoxic T lymphocyte-associated antigen-4 (CTLA-4), which is also known as CD152, is an immune checkpoint that downregulates the immune response and plays a significant role in the pathogenesis of this disease [10].

A class of lymphocytes, the T cells, are vital to the immune system in many conditions and immunity. T helper cells are mediator cells in the immune response. The recognition of pathological microorganisms (i.e., viruses, bacteria, and parasites) that have invaded the body by T lymphocytes is mediated by major histocompatibility complex (MHC) molecules. When they encounter the molecular structures of these microorganisms, major histocompatibility complex molecules are immediately activated and stimulate the secretion of cytokines from B-lymphocytes. Major histocompatibility complex molecules also play a crucial role in the development of Hashimoto’s thyroiditis and other autoimmune thyroid diseases. Indeed, the evidence in the literature suggests that HLA class II molecule expression is increased in the thyroid epithelial cells of patients with Hashimoto’s thyroiditis and Graves’ disease [9]. Some human leukocyte antigen (HLA) types, such as HLA-DR5, are more common in people with Hashimoto’s thyroiditis compared to the general population [8,10]. In addition, a genetic defect in suppressor T cells in Hashimoto’s thyroiditis leads to an impairment in cellular immunity. Suppressor T lymphocytes are unable to suppress the activation of T helper cells during an immune response as a result of this defect. Thyroid gland cells increase the expression of MHC class II antigens on their surface after being stimulated by cytokines, which are produced by lymphocytes due to the interaction between T and B lymphocytes. Current evidence in the literature suggests that cytokines, which play a significant role in the regulation of immune and inflammatory responses, have a crucial role in the pathogenic apoptotic pathways, autoimmunity, and the development of Hashimoto’s thyroiditis [11]. An autoimmune event is assumed to begin with the activation of CD4 (helper) T lymphocytes specific for thyroid antigens. It is unknown how these cells are activated, but there are two hypotheses. The first is the autoimmune hypothesis, which posits that thyroid epithelial cells themselves have intracellular proteins that are specific to helper T cells. This hypothesis is supported by the presence of HLA-DR, HLA-DP, and HLA-DQ molecules in the thyroid cells of patients with autoimmune thyroiditis. It is thought that the atrophic variant of Hashimoto’s thyroiditis is inherited through the HLA-DR3 gene, and the goiter variant is inherited through the HLA-DR5 gene. These molecules are required for antigen presentation to CD4 T cells. Interferon gamma released from T cells has also been shown to increase the expression of MHC class II molecules and to cause a re-stimulation of T cells. It is still unclear how T cells are initially activated in Hashimoto’s thyroiditis patients according to this hypothesis. It has been suggested that suppressor T lymphocyte dysfunction is crucial in the pathogenesis of this phenomenon. It is also known that many stimulating molecules are required for the activation of T cells, apart from HLA antigens. The other hypothesis is the molecular similarity hypothesis. According to this hypothesis, a cross-reaction occurs and activates specific T cells after a viral or bacterial infection containing a protein similar to the thyroid protein. However, there is no evidence to support the molecular similarity hypothesis, as no such virus or bacterial infection has been reported before Hashimoto’s thyroiditis onset.

Cytotoxic T lymphocytes play an essential role in the development of Hashimoto’s thyroiditis. The CTLA-4 gene encodes a costimulatory molecule that suppresses the T cell-mediated immune response and plays a critical role in the maintenance of peripheral immunological self-tolerance, as reported in the literature [12]. On the other hand, the CTLA-4 gene has a vital role in the pathogenesis of autoimmune diseases, such as Hashimoto’s thyroiditis, type 1 diabetes mellitus, and Graves’ disease. Another molecular mechanism involved in the pathogenesis of Hashimoto’s thyroiditis is apoptosis [13]. Apoptosis (programmed cell death) plays a significant role in massive thyrocyte destruction. Incorporation of the Fas receptor with the Fas L ligand initiates apoptosis. In Hashimoto’s thyroiditis, there is an overproduction of Fas and Fas L ligands, which causes increased apoptotic figures in the thyroid tissue. Fas molecule is not present in the thyroid cells of healthy population, but it is expressed in the thyroid cells of subjects with Hashimoto’s thyroiditis. Additionally, Fas molecule expression is controlled by interleukin 1. Fas ligand has been found at high levels in both normal thyroid cells and the thyroid cells of patients with Hashimoto’s thyroiditis. This finding suggests that abnormal Fas expression leads to the activation of apoptosis in the thyroid cells. The authors reported that Fas expression increases when interleukin-1 is added to the culture of normal thyroid cells, and subsequently, apoptosis begins in most of the cells, resulting in cell death [14].

Antibodies present in HT are thought to cause T cell-mediated thyroid damage. It is known that T helper 1 and T helper 2 pathways play a role in the pathogenesis of autoimmune diseases. Hashimoto’s thyroiditis develops when T helper 1 group members activate CD8+ T cells that are responsible for destroying the thyroid tissue. However, recent studies suggest that the T helper 2 group also plays a role in the pathogenesis of Hashimoto’s thyroiditis. Autoimmune and inflammatory diseases are closely related to the regulatory effects of T helper 2 cytokines.

Cytokines are secretory proteins that function as the mediators of immune and inflammatory reactions. They have a high biological activity that enables the communication between cells. These proteins play significant roles in initiating and maintaining thyroid autoimmunity [15]. Cytokines are secreted by macrophages and NK cells in the innate immune system, while they are typically secreted by T lymphocytes in adaptive immune responses. There is a specific messenger RNA for each cytokine that encodes them. Interestingly, cytokine production and the magnitude of the immune response are genetically controlled by cytokines. Infectious diseases, hematopoiesis, intercellular interaction, cell differentiation and activation, organ development during embryogenesis, and regulation of the immune response are some settings in which cytokines have a role. The relationship between lymphoid, hematopoietic, and inflammatory cells involved in the immune response is also regulated by cytokines. Cytokines regulate mitosis, cell migration, cell differentiation, cell survival, and cell death. IL-6 is a cytokine family member and is an important pro-inflammatory mediator required for the initiation of inflammation and immunological reactions. It is synthesized by many cell types, such as activated endothelial cells, mononuclear phagocytes, and fibroblasts [16]. It stimulates the production of acute phase proteins, the activation of T cells, the growth and differentiation of antibody-forming B lymphocytes, and the thyroid autoimmunity [15]. Via stimulating the JAK/STAT-3 and the Ras/Erk/C/EBP signaling pathways, IL-6 also plays a vital role in controlling the differentiation and activation of T lymphocytes. Specifically, IL-6 modulates the resistance of T cells to apoptosis, causing the activation of T helper cells and managing the balance between regulatory T cells and Th17 cells. Recent findings suggest that the blockade of IL-6 signaling is effective in treating experimental models of autoimmune and chronic inflammatory diseases, such as multiple sclerosis, inflammation-related cancer, asthma, inflammatory bowel disease, diabetes mellitus, and rheumatoid arthritis. IL-6 is also one of the significant NF-kB-dependent cytokines. IL-6 provides the essential signals for differentiating IL-17-producing CD4+ T helpers (Th17) via the JAK-STAT3 pathway. Th17 cells differentiate from CD4+ T cells via IL-6, IL-1, TGF β, and IL-21. Th17 cells have inflammatory effects that are responsible for autoimmunity. Studies using animal models and humans have shown that Th17 cells are particularly crucial for HT. After inducing the development of autoimmune thyroid disease with iodine in non-obese diabetic H2(h4) mice, which is a spontaneous animal model for HT, it was shown that Th17 and Th1 cells increased in the spleen and the thyroid gland of the mice [17].

The characteristics of HT include increased serum levels of thyroid autoantibodies and typical sonographic features. A thyroid test panel may reveal thyrotoxicosis at the early phase of the disease due to the immune destruction of the thyroid tissue. However, patients may also be euthyroid or hypothyroid.

Approximately 95% of the cases are women, as this disease is seen 8–10 times more often in women than men [18]. Hypothyroidism can cause systemic inflammation at the cellular level in the body, and this alteration has been linked to metabolic diseases and some types of cancer. [19,20,21]. A novel marker, platelet/lymphocyte ratio (PLR), is a measure that can be obtained at a lower cost than a complete blood count and has been shown to be increased in various medical conditions [22,23,24]. In inflammatory disorders that stimulate the bone marrow, there is an increase in the platelet-to-lymphocyte ratio (PLR) due to the decrease in the number of lymphocytes while platelets are produced [25,26]. Thus, it is obvious that the PLR is elevated in inflammatory conditions.

In the present work, we aimed to evaluate the PLR, which is one of the markers of systemic inflammation, in patients with an established diagnosis of Hashimoto’s thyroiditis.

## 2. Methods

Patients with a diagnosis of HT were enrolled in this study. The subjects were presented to the outpatient internal medicine clinics of our institution between January 2022 and October 2022. The local ethics committee approved the study (approval no: 2022/139). The subjects with HT were divided into two groups based on their thyroid test results: euthyroid and hypothyroid-thyrotoxic. Subjects who visited the outpatient clinics for a routine check-up and were deemed healthy after their assessment were enrolled as the controls. Subjects with pregnancy, cancer, active infection, and hematologic disorders were excluded.

The characteristics of the participants, such as age, sex, and laboratory parameters, including thyroid-stimulating hormone (TSH), free T4 (fT4), C-reactive protein (CRP), aspartate transaminase (AST), alanine transaminase (ALT), white blood cell count (WBC), lymphocyte count (lym), hemoglobin (Hb), hematocrit (Hct), and platelet count (Plt), and the levels of the study population were obtained from the institutional database and recorded. The PLR was determined by dividing the platelet count by the lymphocyte count. All laboratory test results were from the time of the initial diagnosis of HT. The data of the euthyroid HT, hypothyroid-thyrotoxic HT, and control groups were compared.

### Statistical Analyses

Statistical analyses were performed using the SPSS statistics software (SPSS 16.0 for Windows, IBM Co., Chicago, IL, USA). The Kolmogorov–Smirnov test was used to evaluate the normality of the study variables. Since none of the study variables fit into the normal distribution, the Kruskal–Wallis test was applied to compare the study variables. These parameters were reported as median (min–max). Post hoc analysis was performed using Tukey’s test. Categorical variables were compared using the chi-squared test and reported as numbers and percentages. A Pearson’s correlation test was used to assess the correlation between the study variables. A regression analysis considering age and other variables was performed to observe whether the PLR was an independent marker of HT. A *p*-value of less than 0.05 was considered statistically significant.

## 3. Results

The study population included 196 subjects: 66 in the euthyroid Hashimoto’s thyroiditis group, 62 in the hypothyroid-thyrotoxic Hashimoto’s thyroiditis group, and 68 in the control group. The median age of the euthyroid Hashimoto’s thyroiditis, the hypothyroid-thyrotoxic Hashimoto’s thyroiditis, and the control groups were 45 (22–71) years, 44 (26–77) years, and 36 (26–58) years, respectively (*p* < 0.001). The post hoc analysis revealed that the age of the euthyroid Hashimoto’s thyroiditis group was not significantly different from the age of the hypothyroid-thyrotoxic Hashimoto’s thyroiditis group (*p* = 0.052). However, the control subjects were significantly younger than both the euthyroid Hashimoto’s thyroiditis group (*p* = 0.04) and the hypothyroid-thyrotoxic Hashimoto’s thyroiditis group (*p* < 0.001). A total of 60 patients (91%) from the euthyroid Hashimoto’s thyroiditis group, 54 (87%) from the hypothyroid-thyrotoxic Hashimoto’s thyroiditis group, and 53 (78%) from the control group were women (*p* = 0.09).

There was no significant difference between the three study groups according to AST (*p* = 0.66), ALT (*p* = 0.42), and WBC (*p* = 0.24). However, the median levels of Hb (*p* = 0.02), Hct (*p* = 0.02), Plt (*p* < 0.001), lym (*p* < 0.001), CRP (*p* < 0.001), TSH (*p* < 0.001), and fT4 (*p* < 0.001) were significantly different between the study groups. The characteristics and data of the study population are shown in Table 1.

The median PLR for the euthyroid Hashimoto’s thyroiditis group, the hypothyroid-thyrotoxic Hashimoto’s thyroiditis group, and the control group were 137% (69–272), 177% (72–417), and 103% (44–243), respectively. The post hoc analysis revealed that the PLR of the euthyroid Hashimoto’s thyroiditis group was significantly higher than the PLR of the controls (*p* < 0.001). The PLR of the hypothyroid-thyrotoxic Hashimoto’s thyroiditis group was significantly increased compared to both the euthyroid Hashimoto’s thyroiditis group (*p* = 0.02) and the control group (*p* < 0.001).

**Figure 1:**
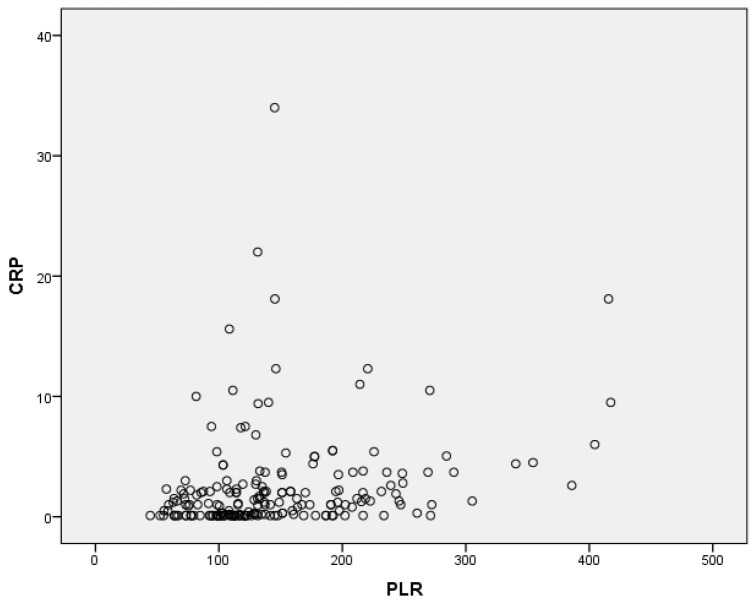
Correlation between CRP and PLR.

In the correlation analysis, there was a significant positive correlation between the CRP and the PLR levels (*r* = 0.24, *p* = 0.001) (Figure 1).

The binary logistic regression analysis, which considered TSH, age, and CRP, as well as the PLR, revealed that the PLR was an independent marker for HT (*p* < 0.001, OR: 1.2, 95%CI: 1.01–1.04).

## 4. Discussion

The most important outcomes of the present study are as follows: (a) the PLR of the subjects with increased Hashimoto’s thyroiditis is significantly higher than the PLR of the control group, and (b) there is a strong positive correlation between the PLR and CRP.

As an autoimmune disorder, Hashimoto’s thyroiditis causes systemic inflammation at the micro level in the body, which leads to hypothyroidism and an enlargement of the thyroid gland. Hashimoto’s thyroiditis is a common cause of hypothyroidism and goiter, especially in territories where iodine deficiency is not endemic [27]. The pathophysiology of Hashimoto’s thyroiditis is related to the activation of T-cells, HLA, DR3, DR4, DR5, and multiple genetic factors. However, its etiology may also be related to iodine intake, various viral infections, and different medications. This has been supported by numerous studies [28,29]. These data suggest the role of inflammation in Hashimoto’s thyroiditis. Additionally, the PLR has been associated with inflammatory conditions, such as type 2 diabetes mellitus [30], irritable bowel disease [31], hepatosteatosis [32], infections [33], and cancer [34]. Therefore, it is not unexpected to find elevated PLR in HT. Accordingly, increased PLR levels were noted in the thyroiditis patients compared to the control subjects in the present study. Moreover, the PLR of the thyroiditis patients was even higher in the hypothyroid/thyrotoxic group compared to the euthyroid Hashimoto’s thyroiditis subjects.

Recent literature suggests that the PLR is a valuable marker of inflammation and a diagnostic or prognostic index for certain conditions, such as cancer [35]. In our study, we found elevated PLR levels in Hashimoto’s thyroiditis patients (either euthyroid or hypothyroid-thyrotoxic Hashimoto’s thyroiditis) compared to the control subjects. Additionally, there was a positive correlation between CRP and the PLR in the HT patients. Some studies have reported high PLR levels, low lymphocyte counts, and high platelet counts in subjects with Hashimoto’s thyroiditis; this is similar to our results, which show that the PLR is higher in the Hashimoto’s thyroiditis patients compared to the control subjects [36]. Increased PLR levels in HT could be related to the autoimmunity-triggered T lymphocyte stimulation. Our study demonstrates that the PLR is a useful index in the detection of autoimmune diseases and inflammation, as it is statistically significantly higher in the Hashimoto’s thyroiditis patients compared to the controls. In addition, we found a positive correlation between CRP and the PLR in our study. This relationship is consistent with the well-known link between autoimmune thyroid diseases and CRP levels.

Platelets participate in clot formation but also have other functions in the body. Elevated platelet levels have been reported in infections. Mishra et al. showed that severe bacterial infections were characterized by high platelet count levels [37]. Moreover, Harris et al. found that increased levels of circulating thrombocytes could be a marker of surgical site infection in subjects that have undergone head and neck surgery [38]. Infections are associated with a high inflammatory burden, as HT is. We can explain the higher platelet counts in the HT subjects through this phenomenon. Not only infections but also inflammatory conditions have been reported to be associated with thrombocytosis. Patients with Behcet’s disease may present with increased platelet count levels in the blood [39]. In addition, increased thrombocyte counts in subjects with rheumatoid arthritis were noted in a study by Hutchinson et al. [40]. It is clear that Hashimoto’s thyroiditis is an inflammatory condition. Therefore, the same inflammatory pathways may induce platelet production in HT subjects. Following the literature data, we found elevated platelet count levels in the HT patients compared to the healthy control subjects in the present study.

Systemic inflammation is characterized by a low lymphocyte count in the blood [41]. A decrease in lymphocyte count is associated with poor prognosis in various conditions [42]. According to the CALIBER cohort study’s results, low levels of lymphocyte count were associated with heart failure and mortality [43]. According to a study by Fathi et al., COVID-19, also known as the millennium pandemic, is characterized by a decrease in lymphocyte count, particularly in severe cases [44]. Iseki et al. evaluated the lymphocyte count values of 362 colorectal cancer patients and found that overall survival was significantly lower in the subjects with low lymphocyte counts [45]. Lymphocyte count was also proposed as a predictor of nutrition. The authors analyzed the lymphocyte levels of 912 women and reported that a low lymphocyte count should be considered a marker of malnutrition in women [46]. Lymphocyte count is a prognostic factor in patients with pulmonary conditions. Acanfora et al. followed an older cohort with chronic obstructive pulmonary disease for three years in a prospective study and found that lymphocyte count was inversely correlated with mortality in those subjects [47]. Following the literature data, we found lower lymphocyte count levels in the patients with Hashimoto’s thyroiditis in the present study. Interestingly, the lymphocyte count of the hypothyroid/thyrotoxic HT patients was even lower than that of the euthyroid HT subjects.

We speculate that there may be a causal relationship between elevated PLR and HT. It is well known that inflammation and infection cause an overproduction of thrombocytes, leading to increased platelet numbers in the bloodstream. This could explain why the PLR is raised in inflammatory conditions, such as HT [48,49]. HT is an inflammatory condition; in this way, an elevation in platelet count is expected, even within the normal range. Lymphopenia, or a low level of lymphocytes, is also commonly seen in inflammatory conditions [50]. Therefore, a combination of elevated platelet and decreased lymphocyte counts can lead to increased PLR levels. Indeed, the platelet levels of the hypothyroid/thyrotoxic HT group were higher than those of the euthyroid HT and control groups. Moreover, the lymphocyte levels of the hypothyroid/thyrotoxic HT group were lower than those of the euthyroid HT and control groups in the present study. As a result, the highest PLR was noted in the hypothyroid/thyrotoxic HT subjects.

Using a specific marker for Hashimoto’s thyroiditis is essential in terms of making early and accurate diagnosis and preventing possible complications of Hashimoto’s thyroiditis as a result of hypothyroidism. These complications can be listed, such as heart diseases [51], depression [52], birth defects [53], dyslipidemia [54], and Hashimoto encephalopathy [55]. The PLR could provide useful diagnostic value as an additional diagnostic tool in the establishment of HT diagnosis at the earliest time, which can decrease the rate of HT complications.

There are also studies in the literature that report opposite findings. For example, the authors found decreased PLR levels in the HT subjects compared to the controls in a recent work [56]. However, the PLR levels were higher in the patients with HT than those in the healthy controls in our study.

There are several limitations to our study. First, the retrospective design may introduce selection bias, which can affect the reliability of the findings in the present study. Second, the study cohort was relatively small, which limits the scientific value of our results. Third, the single-center nature of the study may limit the generalizability of our results.

## 5. Conclusions

Our study indicates that increased PLR levels can be a useful additional diagnostic tool in patients with HT. We think that the PLR, as an inexpensive tool, may yield significant diagnostic contribution in HT.

## Figures and Tables

**Table 1:** Laboratory data of the study cohort.

	Euthyroid HT Group (*n* = 66)	Hypothyroid-Thyrotoxic HT Group (*n* = 62)	Control Group (*n* = 68)	*p*
Age (years)	45 (22–71)	44 (26–77)	36 (26–58)	<0.001
AST (U/L)	18 (10–55)	17 (11–36)	17 (6–43)	0.66
ALT (U/L)	17 (6–54)	15 (7–68)	17 (7–45)	0.42
WBC (k/mm^3^)	6.8 (4.2–10.9)	6.8 (4.3–10.2)	7.4 (3.2–9.2)	0.24
lym (k/mm^3^)	1.9 (1–4.5)	1.6 (0.6–3.6)	2.1 (0.7–4.6)	<0.001
Hb (g/dL)	13.6 (10.6–16.5)	13.3 (9.5–15)	13.7 (12.1–17)	0.02
Htc (%)	42 (33–50)	41 (29–46)	41 (36–53)	0.02
Plt (k/mm^3^)	282 (154–512)	271 (141–409)	205 (153–405)	<0.001
CRP (mg/L)	1 (0.1–22)	3.1 (0.1–18.1)	0.2 (0.1–3.4)	<0.001
TSH (uIU/mL)	1.7 (1–3.9)	6.5 (4.2–58)	1.4 (0.5–4.2)	<0.001
fT4 (ng/dL)	1 (0.8–3.1)	0.9 (0.6–1.1)	0.9 (0.7–1.2)	<0.001
PLR (%)	137 (69–272)	177 (72–417)	103 (44–243)	<0.001

## Data Availability

Data will be made available by the corresponding author upon reasonable request.
